# Effectiveness of Tai Chi Yunshou motor imagery training for hemiplegic upper extremity motor function in poststroke patients: study protocol for a randomized clinical trial

**DOI:** 10.1186/s13063-022-06283-z

**Published:** 2022-04-21

**Authors:** Lin Hong Jiang, Li Juan Zhao, Yang Liu, Hong Zhang, Si Cong Zhang, Wei Qin Cong, Rui Qi

**Affiliations:** 1 Shanghai University of Traditional Chinese Medicine Yueyang Hospital of Integrated Traditional Chinese Medicine and Western Medicine, Shanghai, 200437 China; 2 Shanghai University of Traditional Chinese Medicine, Shanghai, 201203 China

**Keywords:** Tai Chi Yunshou, Motor imagery training, Upper extremity motor function recovery, Stroke, Rehabilitation

## Abstract

**Background:**

Evidence concerning the effect of Tai Chi Yunshou motor imagery training (TCY-MIT) on upper extremity motor function (UE-MF) recovery in poststroke patients is lacking, and few studies have examined the neural mechanisms of MIT. The study was designed to assess the effectiveness of TCY-MIT and its possible neural mechanisms.

**Methods/design:**

The study is an assessor-blinded, parallel, superiority, randomized clinical trial. A total of 78 eligible participants will be randomly assigned to 2 groups in a 1:1 ratio. Participants in the control group will receive (conventional rehabilitation therapies) CRTs for 40 min per day, 6 days per week, for 3 weeks. Participants in the intervention group will receive CRTs combined with TCY-MIT (30 min per day, 6 days per week, for 3 weeks). The primary outcome measure is the Fugl-Meyer Assessment of Upper Extremity. Secondary outcome measures are the Box and Block Test, muscle strength test, modified Barthel index, and Pearson correlation coefficients. All outcomes will be assessed at baseline, after completion of the intervention (1, 2, and 3 weeks), and at the end of follow-up (2 months). The outcome assessor will be blinded to the group allocation of the participants.

**Discussion:**

We expect this assessor-blinded, parallel, superiority, randomized clinical trial to explore the effectiveness of TCY-MIT combined with CRTs compared with CRTs alone for UE-MF in poststroke patients.

**Trial registration:**

Chinese Clinical Trial Registry ID: ChiCTR2100048868. Registered on 19 July 2021

**Supplementary Information:**

The online version contains supplementary material available at 10.1186/s13063-022-06283-z.

## Background

Approximately 85% of stroke survivors undergo varying degrees of upper extremity motor dysfunction [1]. Patients with residual upper limb motor deficits have poor performance in daily life activities, and more than half of them do not fully functionally recover within 6 months [2]. Some clinical rehabilitation therapies have certain requirements for patients’ muscle strength, professional guidance training and grounds, which prolong the time before a large proportion of stroke patients with upper limb dysfunction can return to family and society [3]. These results drive us to find rehabilitation therapies that are more effective and easier to handle for patients participating.

Tai Chi is a martial arts form that originated in China. It is practised in many Asian communities and has growing popularity in Western countries [4]. Practising Tai Chi improves poststroke patient independence and quality of life by relaxing joint tension, improving joint mobility, and promoting motor coordination [5]. Tai Chi Yunshou (TCY) exercise is the basic technology form of Tai Chi [6], and its motion trail is like a cloud moving through the sky, continuously without end, which includes the linkage model of upper limbs, trunk, and lower limbs [7]. Some randomized controlled trials (RCTs) have shown that TCY can effectively improve the upper limb motor function of poststroke patients [8, 9]. However, TCY exercise has certain requirements for the level of motor function of patients and professional guidance. Therefore, there is a risk that stroke patients’ ligaments and joints will be damaged when motor function and muscle strength are not in accordance with this training or with no professional guidance, so its clinical application is limited [10].

In recent years, a novel and active mental practice called motor imagery training (MIT) has emerged [11], which has been applied to all stages of stroke rehabilitation [12] and is easy to learn and master without restrictions on actual movement and training sites [11]. A meta-analysis of RCTs investigating the benefits of MIT for upper extremity motor dysfunction concluded that the clinical efficacy of the evidence supports MIT improving motor function of the upper limbs in stroke rehabilitation.

To address poststroke patients’ difficulty in performing TCY exercise, this study was designed to investigate the effectiveness of TCY combined with MIT on upper extremity motor function (UE-MF) recovery in these patients. Furthermore, there is minimal brain plasticity evidence associated with this training form. Our objective was also to explore the cortical reorganization patterns after Tai Chi Yunshou motor imagery training (TCY-MIT) in stroke patients. In this study protocol, we will describe the rationale, design, and analytic methods of an RCT designed to investigate the rehabilitative effects of Tai Chi among hospitalized stroke patients in the recovery phase. We hypothesized that poststroke patients with hemiplegic upper extremities have a better treatment and response to TCY-MIT combined with conventional rehabilitation therapies (CRTs) than individuals with CRTs treatment. Meanwhile, there will be more effective connections between damaged brain areas or between normal brain areas and damaged brain areas in the TCY-MIT combined with CRTs group.

## Methods/design

### Primary objective and study hypothesis

The primary objective of this clinical trial is to examine the therapeutic efficacy of TCY-MIT for the recovery of UE-MF in poststroke patients. The study hypothesis is that compared to CRTs, TCY-MIT associated with CRTs has a better therapeutic effect on UE-MF. It seems to be a useful complementary therapy for promoting motor function rehabilitation.

### Secondary objective

This study will also present functional near-infrared spectroscopy (fNIRS)-based resting-state imaging as a neuroimaging technique to record spontaneous brain activity for evaluating brain functional rehabilitation and to provide a precise and effective means for TCY-MIT clinical research in poststroke patients.

### Study design

**Fig. 1:**
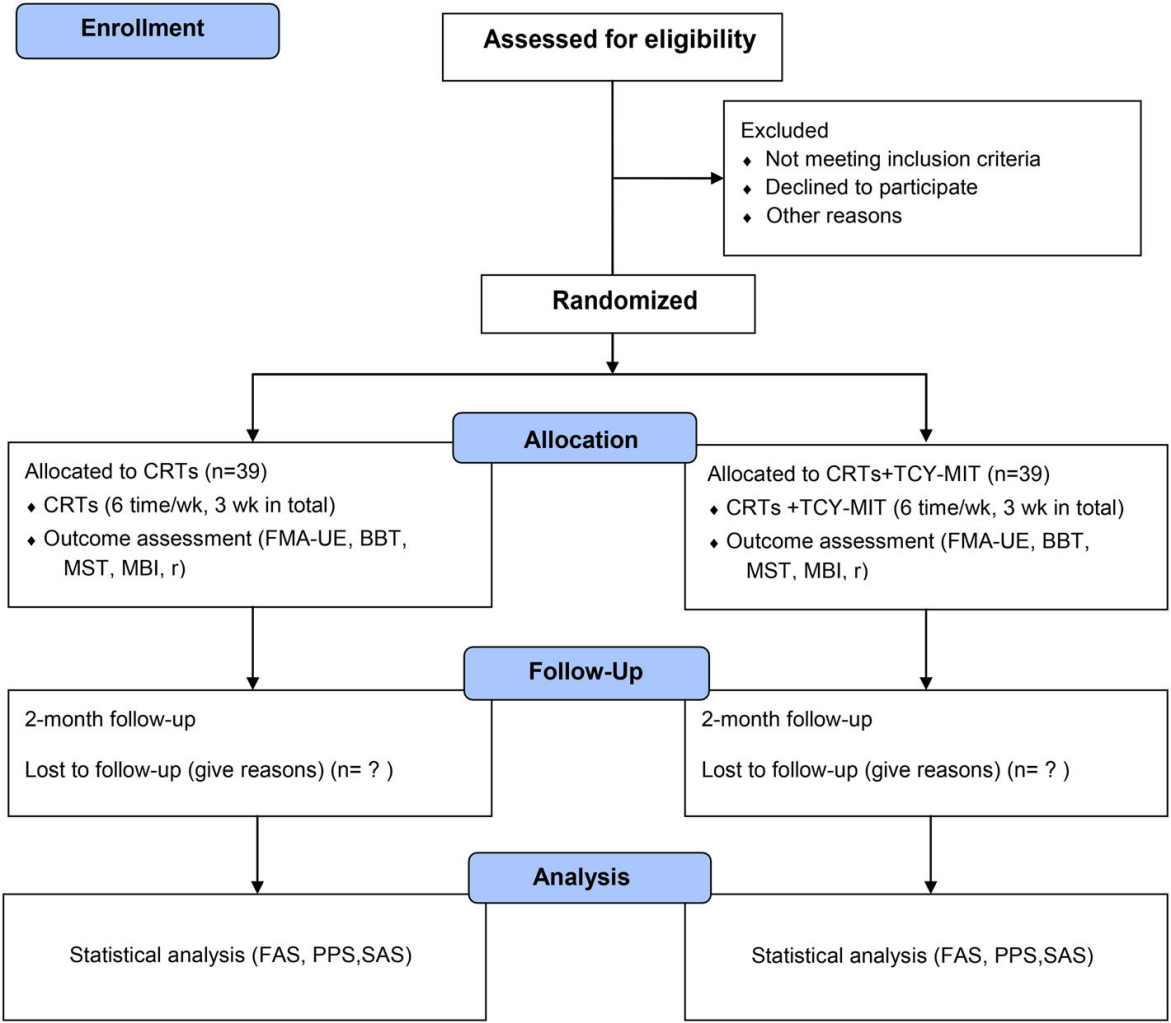
Schematic chart of the study. The template is from the CONSORT 2010 flow diagram. CRTs, conventional rehabilitation therapies; TCY-MIT, Tai Chi Yunshou motor imagery training; FMA-UE, Fugl-Meyer assessment of upper extremity; BBT, Box and Block Test; MST, muscle strength test; MBI modified Barthel index; r, Pearson correlation coefficients; FAS, full analysis set, PPS, per protocol analysis set; SAS, safety assessment set

**Fig. 2:**
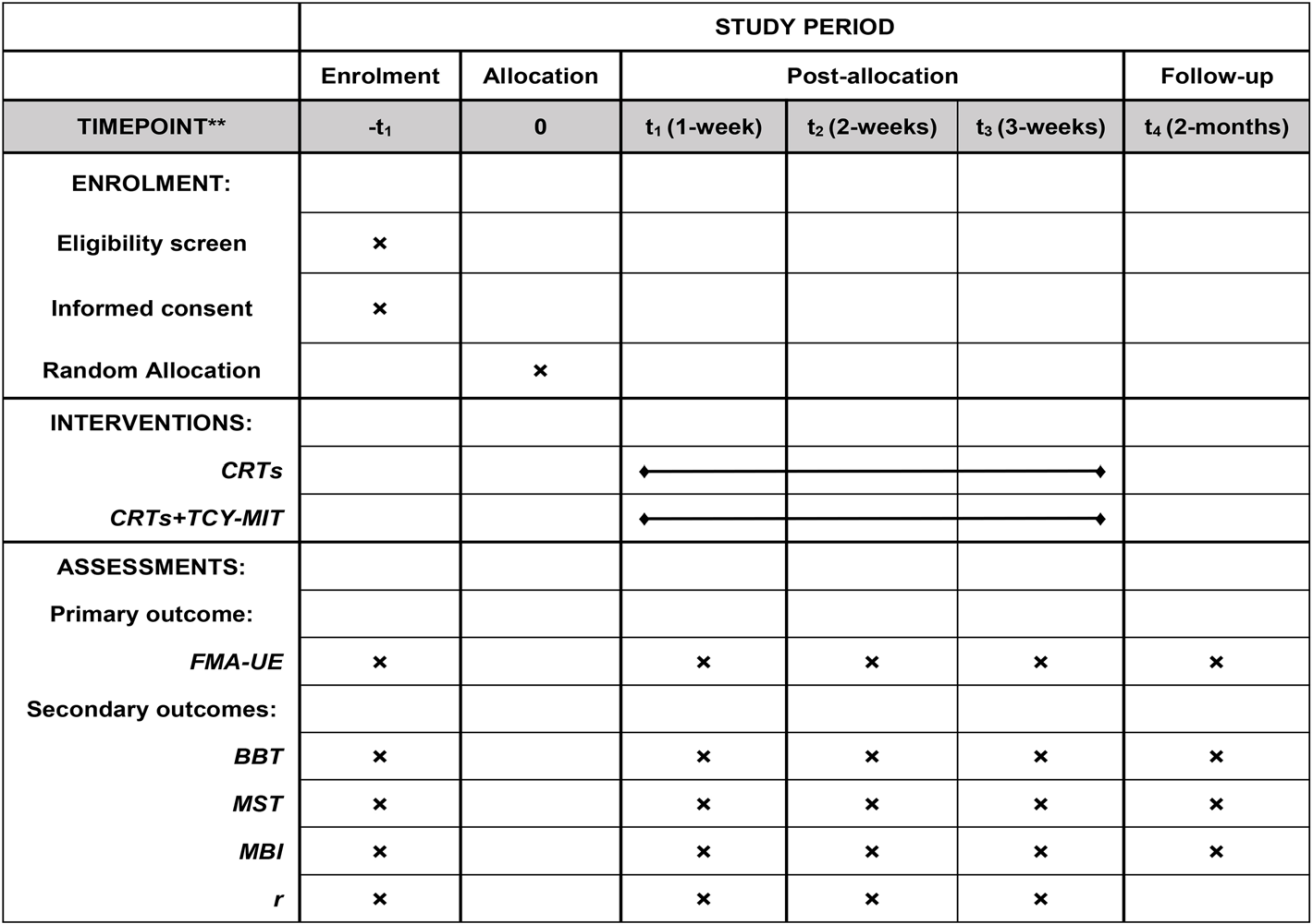
Schematic chart of the study. The schedule of enrolment, interventions, and assessments. The template is from the SPIRIT 2013 statement

This study is an assessor-blinded, parallel, superiority, randomized clinical trial (allocation ratio: 1:1). The design and report of this clinical trial protocol follow the Standard Protocol Items: Recommendations for Interventional Trials (SPIRIT) 2013 statement (Additional file 1) [13]. The schematic chart and study schedule are shown in Figs. 1 and 2.

### Ethical issues

Ethical approval has already been granted by the research ethics committee of Shanghai University of Traditional Chinese Medicine Yueyang Hospital of Integrated Traditional Chinese Medicine and Western Medicine (2021-020, approval received on 8 April 2021). The trial has already been registered with the Chinese Clinical Trial Registry (ChiCTR2100048868, registered on 19 July 2021). Eligible participants will be informed about this trial and sign the consent form before participating in the study.

### Sample size

The sample size was estimated based on a comparison between the intervention group and the control group, represented by the improvement of the Fugl-Meyer Assessment of Upper Extremity (FMA-UE) as the primary outcome. In this study, a two-tailed test was chosen, *α* = 0.05. *μ*_*r*_ and *μ*_*c*_ represent the mean of FMA-LE scores after treatment in the intervention group (TCY-MIT + CRTs group) and the control group (CRTs group) respectively. *K* is the ratio of the sample size between the intervention group and the control group, so *K* = 1. According to the results of previous study [14], *μ*_*r*=_47.00, *μ*_*c*_=36.00, σ = 9, Δ = 3.6, *Z*_1 − *α*_= 1.96, and *Z*_1 − *β*_= 1.28. The calculated sample size of each group was approximately 32 individuals to achieve a power of 0.90 at a significance level of 0.05. After accounting for an anticipated dropout rate of 20%, the total number of patients required for this trial was 78, with 39 in each group. The formulas are as follows:

$$ {\displaystyle \begin{array}{c}{n}_r=\mathrm{K}{n}_c\\ {}{n}_c=\frac{{\left({Z}_{1-\alpha }+{Z}_{1-\beta}\right)}^2{\sigma}^2\left(1+\frac{1}{k}\right)}{{\left({\mu}_r-{\mu}_c-\Delta \right)}^2}\end{array}} $$

where *μ*_*r*_ is the mean of the intervention group, *μ*_*c*_ is the mean of the control group, *K* is the ratio of the sample size between the intervention group and the control group, Δ is the margin of superiority, and σ is the standard deviation.

### Setting and recruitment

This trial will be conducted from August 2021 to April 2022 at the rehabilitation department, Shanghai University of Traditional Chinese Medicine Yueyang Hospital of Integrated Traditional Chinese Medicine and Western Medicine, Shanghai, China. The study will be mainly recruited through WeChat promotions, posters, and promotional leaflets with the inclusion criteria of the trial. A total of 78 participants who meet the eligibility criteria will voluntarily participate in the trial and sign the informed consent form that has been approved by the ethics committee before enrolment.

### Eligibility criteria

#### Inclusion criteria

1. First event of stroke (infarct or haemorrhage) and in line with the diagnostic criteria of cerebrovascular disease formulated by the 4th National Cerebrovascular Academic Conference, confirmed by CT or MRI;
2. No gender limitation, age 40 to 80 years;
3. Poststroke duration 3 to 6 months;
4. For pure motor hemiplegia, the degree of affected upper and hand is Brunnstrom grade II–VI;
5. Good MI ability assessed by the Kinesthetic and Visual Imagery Questionnaire (KVIQ-20) ≥ 25;
6. Right-handed, as determined by the Edinburgh Handedness Inventory;
7. Agreement of the patient or his or her legal guardian (if necessary) to voluntarily participate in the study and signed informed consent. Able to understand and implement rehabilitation training.

#### Exclusion criteria

1. Unable to perform MIT;
2. Unable to communicate or unable to follow commands;
3. Severe speech, attention, hearing, vision, intelligence, mental or cognitive impairment (MMSE < 27);
4. Current participation in any experimental study;
5. Unable to cooperate complete training and evaluation for any reason;
6. Serious primary or secondary diseases, such as severe liver and kidney haematopoietic system disease, severe cardiopulmonary insufficiency, and arrhythmia.

### Randomization and blinding

All eligible participants will be randomly assigned to two groups in a 1:1 ratio. The computer-generated randomization sequence numbers will be generated using the Statistical Package for the Social Sciences (SPSS) 26.0 statistical software. This work will be performed by an occupational therapist not involved in this trial and kept in sealed envelopes. The occupational therapist will assign participants to the two groups according to the random sequence.

To minimize the source of bias after randomization, we will take the following method during this research: conventional rehabilitation therapists and participants will not have any information about the purpose of this study and whether other treatments will be conducted. Meanwhile, TCY-MIT will be guided by another therapist who will not know whether the patients’ participating in other therapies. Furthermore, participants and research personnel do not know the allocated group. The outcome assessor would not be involved in the whole therapy process. Additionally, to examine the success or failure of blinding, an assessor will be evaluated by calculating new blinding index (BI) [15]. They will be inquired which group do they think patients take part in and choose one item from the following answers: CRTs group, TCY-MIT group, or unknown. The new BI is scaled to an interval of − 1 to 1 (0 = consistent with perfect blinding; 1 = complete lack of blinding; -1 = opposite guessing which may be related to unblinding). If a higher-than-expected proportion guesses correctly, a discussion will be made concerning the potential biases causing partial unblinding.

### Interventions

#### Control group

Eligible participants who are assigned to the control group will receive basic treatment, including daily rehabilitation training and clinical medications. Conventional rehabilitation therapies include physical therapy (PT) and occupational therapy (OT). The PT mainly includes functional training, modality, and manual therapy. The OT mainly includes upper limb function training. Those therapies above are the trained therapist based on the participants’ dysfunctional situation to choose the corresponding rehabilitation training, which is to promote upper limb motor function recovery as the main purpose. Specifically, all participants will not receive Tai Chi treatments during the intervention. And therapists are required to track and record specific rehabilitation programs and accurate information such as the frequency and the duration of each intervention and the standard CRTs for 40 min per day, 6 days per week, for 3 weeks.

#### Intervention group

The participants in the intervention group will also accept CRTs, which are identical to the control group. The difference between the two groups is that the intervention group will perform TCY-MIT for 30 min per day, 6 days per week, for 3 weeks. The TCY-MIT consists of 4 steps, and the process is as follows.

**Fig. 3:**
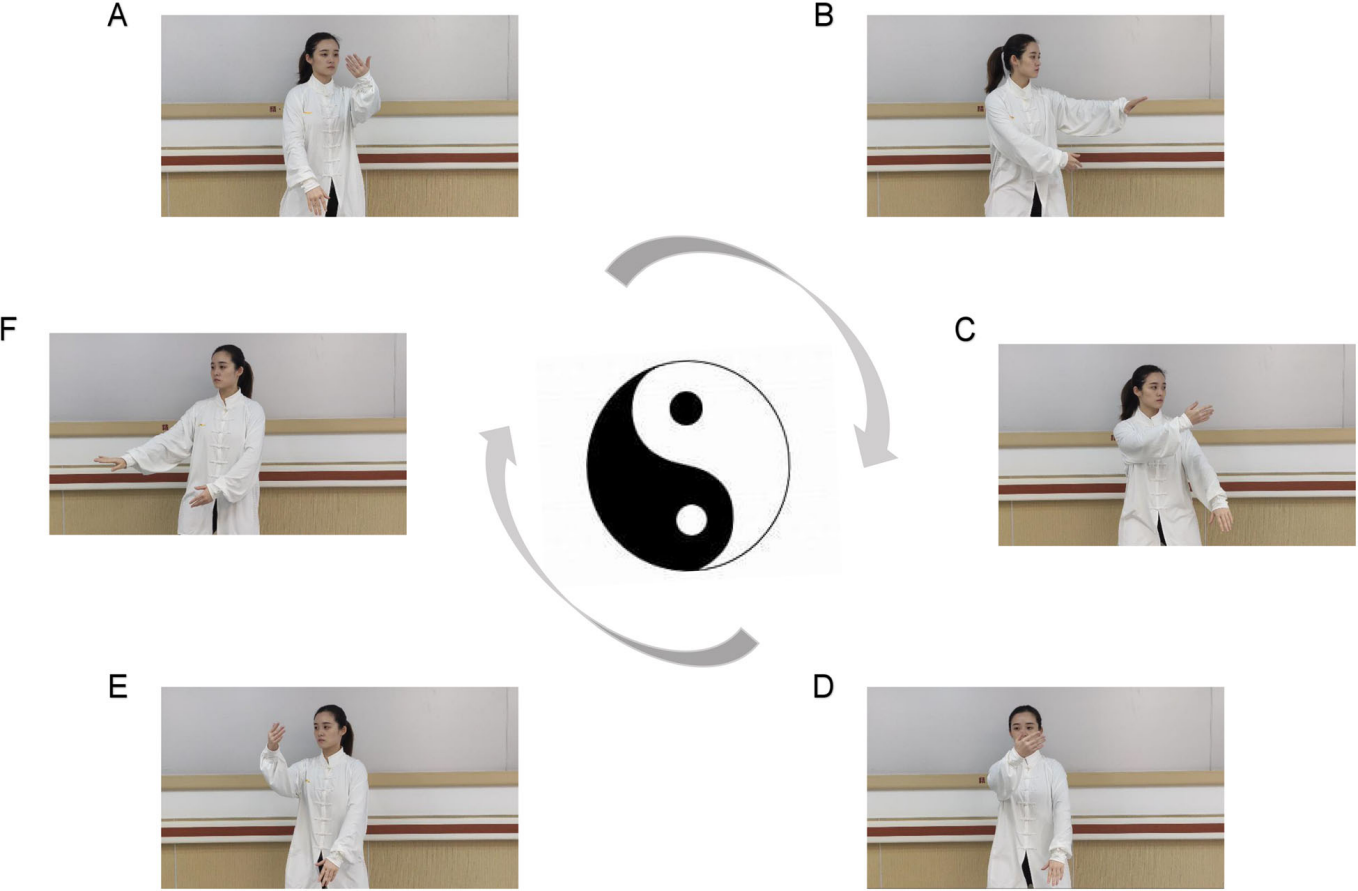
All the movements of Tai Chi Yunshou exercise. The movement of A to F show that how to perform Tai Chi Yunshou exercise

- Step 1: Participants will be organized to watch a video of the TCY exercise movement. The trained TCY therapists will demonstrate the movement of A to F (Fig. 3) and teach the participants to perform TCY exercise with their affected upper limb.
- Step 2: Participants will wear headphones and relax by listening to soft music for 4 min.
- Step 3: Participants will undergo 24 min of imagining the TCY exercise with instructions. Participants will be asked to apply kinesthetic images (first-person images) related to using the affected upper limb.
- Step 4: In the final 2 min, participants will be allowed to refocus on the room.

All TCY-MIT will be administered by a trained occupational therapist. The therapist will check for actual movement and intermittently ask participants to describe what they are doing and what they are experiencing to maintain compliance [16].

### Outcome measures and follow-up

Outcome measures will be performed by a certified, trained occupational therapist who is blinded to the group allocation of the participants. The assessments will be administered at baseline, after completion of the intervention (1, 2, and 3 weeks), and at the end of follow-up (2 months). Furthermore, resting-state fNIRS signal data collection will be a trained fNIRS technician.

### Primary outcome measure

#### Motor function

The primary outcome measure is a change in score on the Fugl-Meyer Assessment of Upper Extremity (FMA-UE). The FMA-UE has been used to assess MIT-induced changes in upper extremity motor impairment in numerous RCTs [17, 18]. The acquired data are in the form of a 3-point ordinal scale (0 = cannot perform at all; 1 = can perform partially; 2 = can perform fully) that is calculated for each item [16]. The scale contains a total of 33 items, which are summed to provide the maximum score of 66. Assessors evaluated all participants immediately before therapy, after 3 weeks of therapy, and at the end of follow-up.

### Secondary outcome measures

The secondary outcome measures are changes in Box and Block Test (BBT), Muscle strength test (MST), modified Barthel index (MBI), and Pearson correlation coefficients (r) from baseline to postintervention follow-up.

#### Manual dexterity

The BBT measures gross manual dexterity in poststroke patients [6]. It consists of a box divided by a partition into two equal-sized compartments and 150 pieces 2.5 cm square of wooden blocks. The participants are requested to use their hemiplegic hands to move the maximum number of wooden blocks without other assistance, and the number of blocks carried from one compartment to the other within 60 s is used as the test score. The participants will be required to repeat the test twice with each hand. After evaluation, the assessor counts the score and takes the average of the two trials as the final score.

#### Muscle strength testing

The computer-assisted (Biometrics Ltd, E-Link) muscle strength test includes hand grip and pinch (thumb and forefinger) strength. Each participant will be asked to grip and pinch the dynamometer with maximal press power. Before this test, the evaluator asks participants if they are ready. Then, the “Go” instruction is given for the participant to start this test. To improve the reliability of muscle strength measurement, participants repeat this test on each hand three times. To minimize deviations and disturbances caused by participants, all participants will not be able to know their MST results during the test (face away from the computer). The examiner counts the score and takes the maximal press power of the three trials as the final score.

#### Activities of daily living

The MBI is a functional outcome measure of activities of daily living (ADL) that includes 10 items; it has satisfactory reliability and validity in poststroke patients. Ten functional activities will be evaluated: feeding, bowel control, bladder control, personal hygiene, transfer, dressing, toileting, ambulation, bathing, and stair climbing. The MBI evaluates global disability by measuring the level of the ADL with a score from 0 (complete dependence) to 100 (full independence).

#### Pearson’s correlation coefficient

**Fig. 4:**
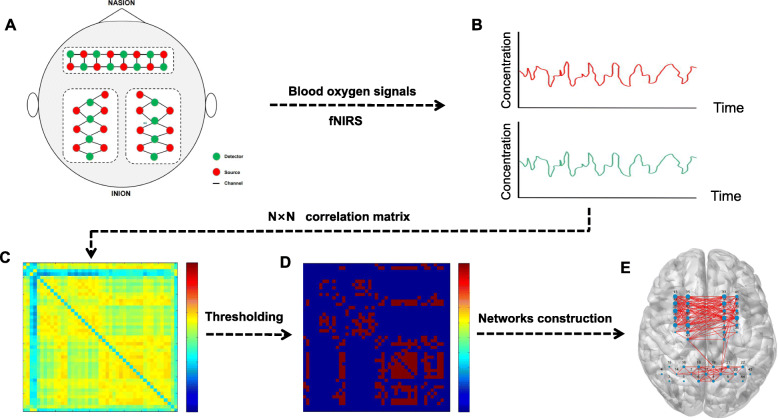
The graph theoretical analysis process of fNIRS-brain networks. **A** Schematic illustration of the fNIRS channels layout. **B** fNIRS-Blood oxygen signals. **C** Construct a N × N correlation matrix. **D** Set threshold to construct a new correlation matrix. **E** Brain functional connectivity

Brain network connection analysis has been widely used in the assessment of impaired brain function [19]. fNIRS is a novel, noninvasive brain function imaging technology to detect changes in oxygenated haemoglobin and deoxygenated haemoglobin in the cerebral cortex in a timely manner [20]. fNIRS will be applied to record resting-state signals with two groups. Brain functional connectivity will be calculated, and graph theoretical analysis will be further applied to represent topological relationships in complex brain networks (Fig. 4).

In this study, we will use a multichannel fNIRS system (NirSmart-II, HuiChuang, China) with two wavelengths (730 nm and 850 nm) at a sampling rate of 11 Hz. The NirSpark software (v1.7.3) package will be used to preprocess fNIRS signal data and construct brain functional networks. We will explore the structural characteristics of the resting brain network in poststroke patients and assess differences between the two groups. Resting-state fNIRS data will be collected for 10 min to subsequently construct brain functional networks. Pearson correlation of oxygenated haemoglobin on time series of each channel and brain region will be calculated, and Pearson’s correlation coefficient (r) is defined as the resting-state functional connection of corresponding channels and brain region strength.

### Data collection and management

Case report forms (CRFs) will be drafted to record original data collection regarding clinical information and assessment of outcomes. Paper copies of the CRFs will be filled in by the outcome assessors who are blinded to the group allocation of the participants. Microsoft Excel 2020 and EpiData 3.1 software will be used to manage data. The original clinical information and assessment of outcomes will be entered into EpiData. Double data entry and verification will be performed by two independent researchers to ensure the truthfulness and accuracy of the data. If there is an inconsistency of data input or missing data, the original participants’ data will be read again to further verify, and the verified data results will be exported to Excel.

### Statistical analysis

A sample size of 64 participants (32 per group) was determined to provide 90% power (*α* 0·05) to detect a mean FMA-UE difference of 11.0 (SD 9) points between the two treatment groups. The main analysis of this trial consists of a comparison of the outcomes after 3 weeks and at the end of follow-up (2 months). The statistician who is blinded to the group allocation of the participants will lock all data before statistical analysis. All data will be analysed with Social Sciences (SPSS) version 26.0 software (IBM Corporation, Armonk, USA) and GraphPad Prism version 9.0 software (GraphPad Software Inc., San Diego, USA). The baseline data and outcome analyses will be conducted on the full analysis set (FAS), using intent-to-treat (ITT) samples and completer samples. The per protocol set (PPS) will be used to analyse effective cases based on FAS. Furthermore, safety analysis will be performed on safety assessment set (SAS). Demographic data will be analysed using a two-sample *t*-test or Wilcoxon rank-sum test for continuous variables and the chi-squared test or Fisher’s exact test for categorical variables. For outcome, variables will be presented as the mean ± standard deviation ($\overline{x}\pm \mathrm{s}$). Primary outcomes and secondary outcomes will be analysed using multivariable analysis of variance (ANOVA) for repeated measures, followed by Tukey's post hoc test. Statistical significance will be set at a *P value* less than 0.05.

## Discussion

Previous studies have investigated the effects of actual TCY on the recovery of upper extremity motor dysfunction in poststroke patients [8, 9]. Scholars have found that kinesthetic motor imagination and visual motion imagination can both mobilize cortical reorganization and neuronal excitability, which could cause structural and functional remodelling, thereby promoting behavioural recovery [21–23]. Hence, we will combine MIT, converting actual TCY to kinesthetic motor imagination that fits poststroke patients with hemiplegia who cannot move or have severely impaired movement in their affected upper limb. With the development of neuroimaging, many studies use objective data to evaluate and predict the prognosis of poststroke patients with damaged brain function [24, 25]. We chose cerebral cortex blood oxygen changes to assess neural activity and regulation of the cerebral cortex after this combination therapy because compared with other neuroimaging activity collection methods, fNIRS is more convenient and has a wider range of applications [26, 27]. All of the clinical procedures will be performed in the hospital, and this treatment will be done with professional guidance. Therefore, the use of TCY-MIT combined with CRT to treat poststroke patients’ upper extremity motor dysfunction is available and safe under this setting. Our hypothesis is that UE-MF, manual dexterity, finger muscle strength, and activities of daily living will be better improved after TCY-MIT combined with CRTs compared with CRTs alone. More effective brain network connections between damaged brain areas or between normal brain areas and damaged brain areas were observed in the TCY-MIT combined with CRTs group, which is based on fNIRS resting-state collection. If the results are as anticipated, this will promote the understanding of the neurological activity of TCY-MIT in poststroke patients. In addition, combination therapy could be widely adopted in poststroke communities with dyskinesia to reduce the global burden of stroke.

Our study has several strengths. (1) the design and report of the trial protocol strictly follow the SPIRIT statement, and this study will adhere to CONSORT 2010. (2) The TCY exercise is a household name, group training program. It is easy to conduct TCY-MIT in the stroke population and to improve the participants’ compliance. (3) Few studies have examined neural activity during TCY-MIT. We will capture cerebral cortex blood oxygen changes during neural activities to reveal the potential mechanisms of TCY-MIT combined with CRTs via fNIRS-based resting-state brain network analysis. We hypothesized that compared with the control group, participants in the intervention group will show increased brain functional connectivity and that the *r* value is related to the primary outcome. The fNIRS technician (blinded to the group allocation of the participants) will be assigned to take charge of fNIRS-based resting-state data collection and analysis. (4) In this trial, we are not only focusing on the recovery of motor function after the intervention, but we are also using the MBI score as an outcome measure to assess ADL in poststroke patients after completion of the interventions (3 weeks) and at the 6-month postintervention follow-up (2 months).

However, there are a few limitations. First, the intervention time for stroke patients is only 3 weeks due to the duration limit of hospitalization that is set for the patients. Second, this is a single-centre study. A multicentre, larger sample size is required to prove our objectives. Third, it is difficult to objectively monitor whether the participants are engaging in TCY-MIT rather than other mental activities. Fourth, the study does not use fNIRS to perform analysis of cortical activation with tasks.

In summary, the results of the study will be beneficial to optimize rehabilitation training methods and provide new therapeutic ideas for stroke rehabilitation. Our team will further explore the individualized rehabilitation program of TCY-MIT in future work.

## Trial status

Recruitment for this trial is currently ongoing. And the study will be completed in June 2022.

## Supplementary Information

**Additional file 1.**

## Data Availability

The datasets used and/or analysed after completing the current study will be available from the corresponding author by reasonable requests.

## Abbreviations

ADL: Activities of daily living
ANOVA: Multivariable analysis of variance
BBT: Box and Block Test
BI: Blinding index
CRTs: Conventional rehabilitation therapies
CRFs: Case report forms
CT: Computed tomography
FMA-UE: Fugl-Meyer assessment of Upper Extremity
KVIQ-20: Kinesthetic and Visual Imagery Questionnaire-20
FAS: Full analysis set
fNIRS: Functional near-infrared spectroscopy
ITT: Intent-to-treat
MIT: Motor imagery training
MBI: Modified Barthel index
MST: Muscle strength test
MRI: Magnetic resonance imaging
MMSE: Mini-Mental State Examination
OT: Occupational therapy
PPS: Per protocol set
PT: Physical therapy
RCTs: Randomized controlled trials
r: Pearson’s correlation coefficient
SPIRIT: Standard Protocol Items: Recommendations for Interventional Trials
SPSS: Statistical Package for the Social Sciences
SAS: Safety assessment set
TCY-MIT: Tai Chi Yunshou motor imagery training
UE-MF: Upper extremity motor function
